# B^0^AT1 Amino Acid Transporter Complexed With SARS-CoV-2 Receptor ACE2 Forms a Heterodimer Functional Unit: *In Situ* Conformation Using Radiation Inactivation Analysis

**DOI:** 10.1093/function/zqab027

**Published:** 2021-05-13

**Authors:** Bruce R Stevens, J Clive Ellory, Robert L Preston

**Affiliations:** Department of Physiology and Functional Genomics, University of Florida College of Medicine, Gainesville, FL, 32610, USA; Department of Medicine, Division of Gastroenterology, University of Florida College of Medicine, Gainesville, FL, 32610, USA; Department of Physiology, Anatomy and Genetics, University of Oxford, Oxford, OX1 3PT, UK; School of Biological Sciences, Illinois State University, Normal, IL, 61790, USA

**Keywords:** ACE2, B^0^AT1, neutral amino acid transport, transporter, membrane, intestine, radiation inactivation, 6M17, sodium-dependent transport

## Abstract

The SARS-CoV-2 receptor, angiotensin-converting enzyme-2 (ACE2), is expressed at levels of greatest magnitude in the small intestine as compared with all other human tissues. Enterocyte ACE2 is coexpressed as the apical membrane trafficking partner obligatory for expression and activity of the B^0^AT1 sodium-dependent neutral amino acid transporter. These components are assembled as an [ACE2:B^0^AT1]_2_ dimer-of-heterodimers quaternary complex that putatively steers SARS-CoV-2 tropism in the gastrointestinal (GI) tract. GI clinical symptomology is reported in about half of COVID-19 patients, and can be accompanied by gut shedding of virion particles. We hypothesized that within this 4-mer structural complex, each [ACE2:B^0^AT1] heterodimer pair constitutes a physiological “functional unit.” This was confirmed experimentally by employing purified lyophilized enterocyte brush border membrane vesicles exposed to increasing doses of high-energy electron radiation from a 16 MeV linear accelerator. Based on radiation target theory, the results indicated the presence of Na^+^-dependent neutral amino acid influx transport activity functional unit with target size molecular weight 183.7 ± 16.8 kDa *in situ* in intact apical membranes. Each thermodynamically stabilized [ACE2:B^0^AT1] heterodimer functional unit manifests the transport activity within the whole ∼345 kDa [ACE2:B^0^AT1]_2_ dimer-of-heterodimers quaternary structural complex. The results are consistent with our prior molecular docking modeling and gut–lung axis approaches to understanding COVID-19. These findings advance understanding the physiology of B^0^AT1 interaction with ACE2 in the gut, and thereby contribute to translational developments designed to treat or mitigate COVID-19 variant outbreaks and/or GI symptom persistence in long-haul postacute sequelae of SARS-CoV-2.

## Graphical Abstract

**Graphical Abstract:**
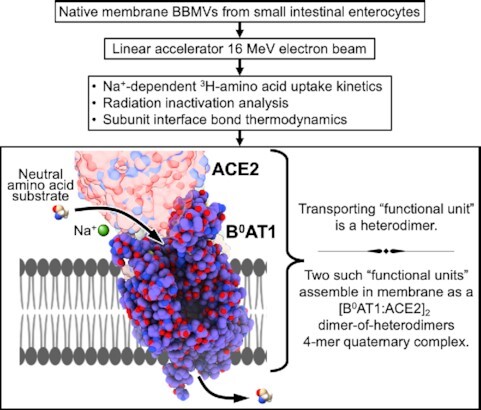

## Introduction

Infection by SARS-CoV-2 requires its receptor binding domain to bind the ectodomain of angiotensin-converting enzyme-2 (ACE2). ACE2 can be stabilized on the surface of plasma membranes by the sodium-dependent neutral amino acid transporter, B^0^AT1, forming an [ACE2:B^0^AT1]_2_ dimer-of-heterodimers quaternary 4-mer structural complex.^1^ Pfizer/BioNTech exploited plasmid constructs of this [ACE2:B^0^AT1]_2_ structure overexpressed in cultured cell membranes^2^ as being crucial to their successful preclinical testing of mRNA candidates encoding SARS-CoV-2 spike protein efficacious vaccine epitopes. Following clinical trials, their ACE2:B^0^AT1-screened choice of BNT162b2 mRNA^2^ was approved by US FDA for emergency use authorization delivery by lipid nanoparticles as the country's first publicly deployed COVID-19 vaccine.

B^0^AT1 (literature aliases: NBB, B, B^0^, B(0)AT1) was originally discovered and functionally characterized by Stevens and coworkers^3–17^ as being the major sodium-coupled neutral amino acid transport system in small intestine villus epithelial cell apical brush border membranes.^7,17^ These seminal studies were obligatory to subsequently assigning the functional properties to an SLC6A19 gene expression product by Broer, Verrey, and colleagues, and in implicating ACE2 as indispensable in epithelial cell trafficking/chaperoning^18^ and apical membrane expression of B^0^AT1.^18–29^ Following recommendations made by Halvor Christensen at a 1994 membrane transport symposium in Stowe, Vermont, the Stevens' NBB (Neutral Brush Border) term^3,7^ was changed to B and then to B^0^, in order to conform to the then-evolving transporter nomenclature convention.^30^ This alluded back to Christensen's pioneering **B**lastocyst classification categories in which the uppercase refers to sodium dependency and the “0” superscript refers to the zwitterion net zero charge of neutral amino acid substrates.^30^ Ultimately, the **NBB/B/B^0^A**mino acid **T**ransporter (AT) various interchangeable appellations in the literature^3–16,30,31^ were eventually consolidated into the current designation “B^0^AT1.”^17,20,32^

The small intestine is the human body's site of greatest magnitude expression of both B^0^AT1 and ACE2.^33–41^ In the mucosa, B^0^AT1 is the central player in villus enterocyte neutral amino acid transport that supplies nutritional amino nitrogen. Its amino acid substrates signal enteroendocrine and goblet cell physiological activities, and steer gut barrier integrity and inflammasome events.^17,25,33,34,42^

Literature reviews/meta-studies published during the period spanning 1990–2010 presaged various pleiotropic physiological roles for B^0^AT1 interactions with ACE2, including the remarkably prescient concept of governing coronavirus infectivity.^17,21,24^ In early 2020, Yan and coworkers in Zhou's group^1^ utilized 2.9 Å resolution cryo-electron microscopy to determine that two B^0^AT1 subunits stabilize two ACE2 subunits in cell membranes as the thermodynamically favored atomic structure [ACE2:B^0^AT1]_2_ multimeric complex that can bind the SARS-CoV-2 spike (PDB ID: 6M17 and PDB ID: 6M18).

SARS-CoV-2 hijacks ACE2 as its receptor in both small intestinal enterocytes and lung pneumocytes.^33,34,43–47^ Pulmonary symptoms are the hallmark of severe COVID-19, while about half of COVID-19 patients manifest extrapulmonary gastrointestinal (GI) tropism with gut clinical symptomology accompanied by virion particles shed in feces and RNA in toilet aerosols in the active phase, and intestinal symptoms persist in long-haul postacute sequelae of SARS-CoV-2 (PASC).^48–53^ The main risk factor decisive for organ-based clinical outcomes of lung versus intestine in COVID-19 is the nature of ACE2 interplay with two particular membrane-bound metalloproteinases—TMPRSS2 and ADAM17—that are expressed in both organs.^54^ These metalloproteinases are responsible for launching the pernicious events of SARS-CoV-2 tropism via their specific cleavage sites on ACE2.^33,34,55,56^ Lung cells do not express B^0^AT1, thus permitting ready access of TMPRSS2 and ADAM17 to pneumocyte monomer ACE2 cleavage sites, resulting in unconstrained lung pathology.^33,34^ However, for enterocytes that can express the [ACE2:B^0^AT1]_2_ complex, our molecular docking studies^55,56^ predicted that the B^0^AT1 subunits sterically interfere with TMPRSS2 and ADAM17 access to the cleavage sites of gut ACE2. Thus, the degree to which B^0^AT1 is expressed and trafficked by ACE2 is likely a pivotal factor that governs gut COVID-19 severity in a given patient. Consequently, the structure–function relationship coupling B^0^AT1 with ACE2 is important to understanding involvement of the intestine in COVID-19 and why some patients are spared yet others are affected. This relationship is poorly understood.

The present study addresses this knowledge gap, in order to provide insights that may lead to developing new therapies and treatments for COVID-19 in current or future outbreaks. Our approach was to exploit radiation inactivation analysis and electron flux density target theory utilizing high-energy ionizing electrons from a 16 MeV linear accelerator. As empirically established by us^57,58^ and others,^59–70^ this technique reveals membrane *in situ* structure–function relationships, accurately identifying the molecular size of “functional units” entwined within physical structures of complex multi-subunit biological systems such as channels, transporters, enzymes, and receptors. We report that sodium-dependent carrier-mediated B^0^AT1 activity *in situ* in small intestinal enterocyte purified apical brush border membrane vesicles (BBMVs) occurs via an apparent physiological “functional unit” of target size molecular weight (mw) ∼184 kDa representing the [ACE2:B^0^AT1] heterodimer components within the ∼345 kDa [ACE2:B^0^AT1]_2_ dimer-of-heterodimer complex.

## Methods

Small intestinal epithelium isolated apical BBMVs were prepared using New Zealand white rabbit ileum mucosa, lyophilized, and reconstituted for use in radiation inactivation experiments as previously described by us.^3,4,7,15,57,58,71^ Briefly, rapidly isolated mucosal scrapings were obtained from 1 m of ileum proximal to the ileocecal junction and treated with 10 mm MgCl_2_, followed by a series of differential centrifugations and progressively diluted washes using 300–10 mmd-mannitol in 1 mm HCl/Tris pH 7.6 buffer. The final pellets were suspended in distilled water using a glass homogenizer. BBMVs (15 mg protein/mL in 100 μL) aliquoted into individual glass ampules were snap frozen in liquid N_2_ and then lyophilized under 20 μm Hg vacuum for 12 h and subsequently stored vacuum-sealed at −10°C until needed for radiation inactivation experiments. For postirradiation uptake assays, the lyophilized BBMVs were reconstituted and equilibrated at 22°C with 100 μL of buffer containing 200 mmd-mannitol in 10 mm HEPES/Tris pH 7.5 followed by 3 passes through a 22-gauge needle.

Lyophilized BBMVs vacuum-sealed in glass ampules were stable for several months at ∼22°C room temperature, such that when reconstituted they displayed >90% of original fresh transport activity and the usual BBMV characteristics observed for fresh BBMVs. As we have published previously,^4,7,15,57,58^ we measured radiotracer labeled l-amino acid or d-glucose time course uptake peak overshoots in zero-*trans* sodium-containing uptake media, and >95% right-side-out sealed spherical compartments ∼1000 Å diameter, with <5% nonsealed pieces of membrane observed in electron micrographs.^4,7,57,58^ Transmission electron microscopy cross-sections were prepared using glutaraldehyde/OsO_4_/uranyl acetate-treated centrifuged pellets of reconstituted lyophilized BBMVs. BBMVs were enriched ∼15-fold in each of the apical membrane markers γ-glutamyl transpeptidase, leucine aminopeptidase, and alkaline phosphatase, relative to mucosal cell scrapings of the starting tissue. On the other hand Na^+^/K^+^ ATPase activity representing basolateral membrane contamination was decreased by ∼70% as previously reported.^4,7,15,57,58^ Alkaline phosphatase (EC 3.1.3.1) activity was employed as a radiation inactivation target size mw internal calibration standard. For each radiation dose, 0.02 mL of reconstituted irradiated BBMV suspension containing 100 mm NaSCN was incubated at 22°C with 1.0 mL 0.9 M diethanolamine pH 9.8, 1.0 mL 30 mm*p*-nitrophenylphosphate (pNPP) in media lacking K^+^ ions, with the *p*-nitrophenol product quantified colorimetrically at 405 nm.^62,66^

Lyophilized BBMVs in thin wall glass ampules under vacuum were irradiated with a high-energy electron beam (16 MeV in a 10 cm uniform beam) delivered by a linear accelerator (Addenbrooke's Hospital, Cambridge, England) over the range of 5–180 kGy in increments of 20 kGy/min or less to prevent sample heating. Samples were fitted in an aluminum block cooled by a dry-ice streaming system. The accelerator was calibrated using Perspex dosimetry. The irradiated vesicles were stored in their vacuum-sealed ampules at −10°C until required for assays. Following postirradiation, BBMVs were reconstituted with 200 mmd-mannitol pH 7.5 buffer as described above, and the vesicles were then allowed to equilibrate for 30 min before transport measurements were made.

Influx initial rates were measured at 22°C in reconstituted BBMVs, defined as the 5 s initial uptake of zero-*trans* (ie, substrate outside but not inside) unidirectional carrier-mediated sodium-dependent portion of total uptake of radiolabeled 0.1 mm [^3^H]-l-alanine or 1 mm [^3^H]-L-serine, as described by us.^3,15,58^ The external vesicle uptake buffer contained either 100 mm NaSCN or 100 mm KSCN in 100 mmd-mannitol pH 7.5. Sodium-dependent carrier-mediated transport activity was calculated from the total radiotracer uptake in Na^+^ media minus diffusion uptake as measured in K^+^ media replacing Na^+^ in the presence of unlabeled 100 mml-methionine or 100 mml-alanine. A rapid mix/rapid filtration apparatus was employed with ice-cold 200 mmd-mannitol stop buffer to arrest uptake, as described by us.^3,15,58^ Uptake measurements were replicated *N* = 6 times.

Radiation inactivation target size mws were obtained by measuring postirradiation remaining activity of zero-*trans* unidirectional sodium-dependent initial influx rates in reconstituted lyophilized BBMVs at various radiation doses:

(1) \begin{eqnarray*} A = {A_0} \cdot {e^{ - kD}}, \end{eqnarray*}

where *A* = activity remaining, *A*_0_ = control initial activity, *D* = radiation dose in kGy units, and *k* = rate constant dependent on target mw. It has been empirically established by us^57,58^ and others^59–70^ that for activity of biological systems in lyophilized preparations irradiated by high-energy electron beams, the "functional unit" radiation target size is calculated by

(2) \begin{eqnarray*} \mathrm{ target}\ \mathrm{ size}\ \mathrm{ mw}\ \ \left( {\mathrm{ kDa}} \right) = 6.4 \cdot {10^3} / {D_{37}}, \end{eqnarray*}

where *D*_37_ = radiation dose (in kGy units) at which activity = *A*_0_^.^*e*^−1^ (ie, 37% of control activity). In practice, target sizes were computed by nonlinear regressions constrained to 100% activity at zero dose radiation, fitting the raw data using the R package “investr” with objects of class “nls” using the function:

(3) \begin{eqnarray*} &&ln\left( {\% \ \mathrm{ remaining}\ \mathrm{ activity}} \right)\\ &&\quad= ln\left( {100} \right) - \left( {\mathrm{ kGy }\cdot \left( {\mathrm{ kDa}\ \mathrm{ target}\ \mathrm{ size}\ \mathrm{ mw}} \right)/6.4 \cdot {{10}^3}} \right). \end{eqnarray*}

Atomic coordinates for PDB ID: 6M18, 6M17, or 6M1D were employed for the molecular structure assemblage of ACE2 subunits with B^0^AT1 subunits as the [ACE2:B^0^AT1]_2_ dimer-of-heterodimers quaternary complex determined by Yan and coworkers in Zhou's group^1^ using 2.90 Å resolution cryo-electron microscopy. In accordance with our previous studies of B^0^AT1 structures,^55,56^ molecular modeling of subunit interactions and interface residues' contact distances were executed using ChimeraX software,^72^ meeting default probe criteria of 1.4 Å or being buried within a 15 Å^2^ area cutoff. Thermodynamics of chain molecular internal and interface energies were computed using PDBePISA.^73^ Molecular structures and their membrane location were generated using PyMOL v2.4.0,^74^ PDBEditor,^75^ ChimeraX,^72^ and Orientations of Proteins in Membranes (OPM) database transmembrane server.^76^

## Results

**Figure 1.**
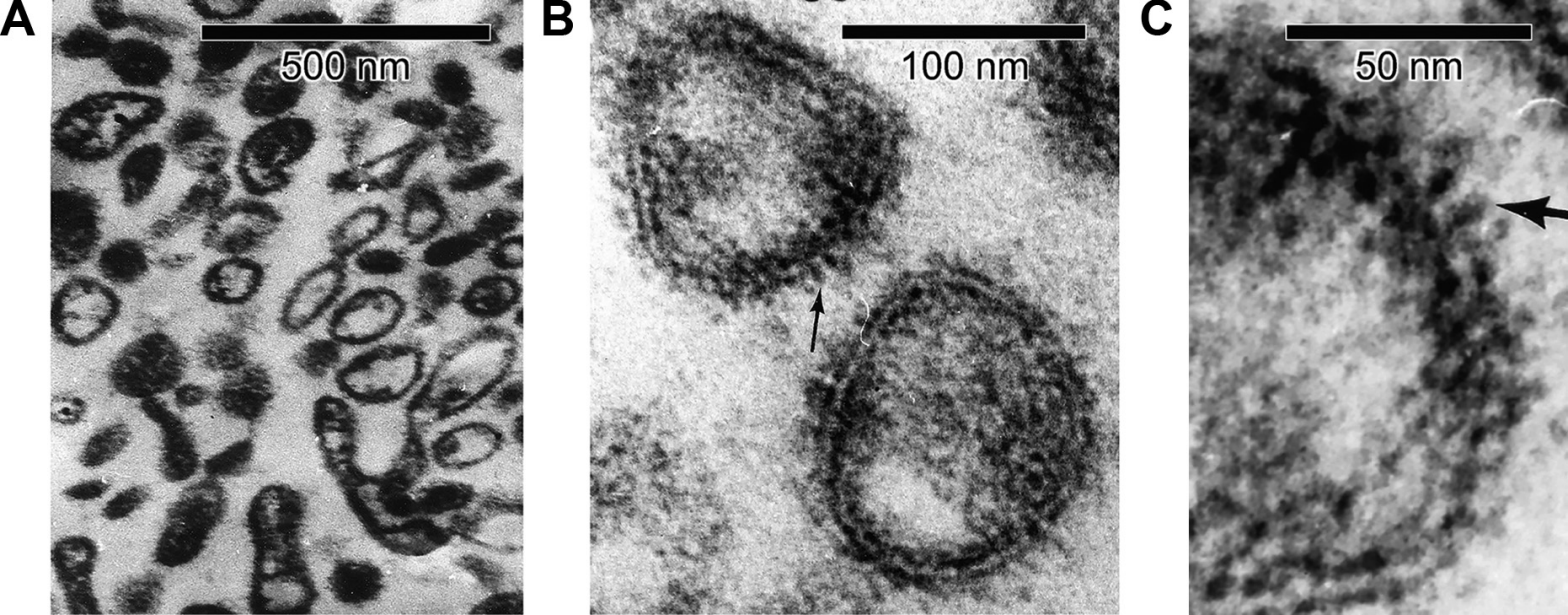
Cross-section Electron Micrographs of Reconstituted Lyophilized Small Intestinal Purified Apical Membrane BBMVs.**(A)** Wide field view of intact BBMV vesicles employed for radiation inactivation of B^0^AT1 functional unit activity, with right-side-out orientation of fuzzy glycocalyx. **(B)** BBMV sealed lipid bilayers showing protruding 100–150 Å knobs (arrow example). **(C)** Close-up view of reconstituted lyophilized BBMV, showing 100–150 Å protruding glycoprotein knobs from membrane surface lipid rafts (arrow example).

Figure 1 shows cross-section electron micrographs of the reconstituted lyophilized small intestinal purified apical BBMVs, which were ∼100 nm diameter. In Figure 1A and B, note the sealed right-side-out BBMVs populated by 100–150 Å protruding knobs from the membrane surface lipid rafts. Such sealed vesicles are essential for measuring uptake of radiotracer substrates across the purified membrane proteophospholipid components that partition a defined space trapping the radiotracer.

**Figure 2.**
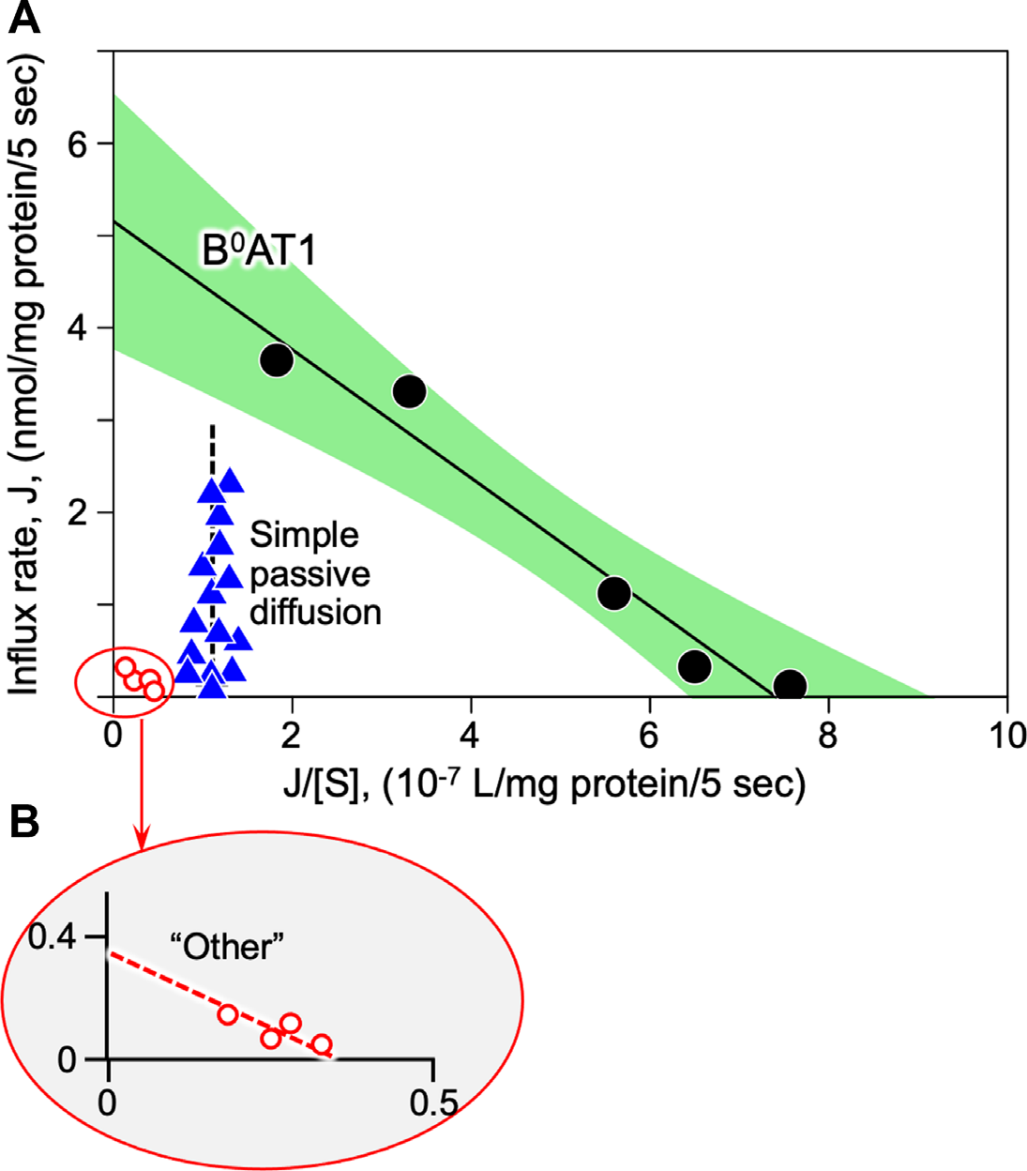
Eadie–Hofstee Plot of Initial Rate Radiotracer Amino Acid Influx Transport Kinetics.**(A)**Employing multivariate nonlinear analyses of zero-*trans* unidirectional [^3^H]-l-alanine initial influx rates measured in reconstituted lyophilized intestinal BBMVs, a single linear component B^0^AT1 (black circles) was derived by subtracting simple passive diffusion (blue triangles) from total l-alanine uptake in Na^+^ media. The B^0^AT1 component fit saturable kinetics per eqn (1) (see the Methods section), defining >95% of the Na^+^-dependent carrier-mediated uptake, as represented by the solid line within green 95% CI. The computed passive diffusion permeability coefficient, *P*, of eqn (1) (abscissa intercept of vertical dashed line) was also independently empirically verified by measuring uptake in K^+^ media replacing Na^+^ in the presence of 100 mml-alanine and/or 100 mm unlabeled methionine. **(B)** An apparent additional trivial carrier-mediated component, labeled “Other” (red open circles), contributed <5% of total Na^+^-dependent maximum uptake activity, and was dropped from subsequent considerations.

The reconstituted lyophilized intestinal BBMV zero-*trans* uptake kinetics exhibited a singular saturable carrier-mediated sodium-dependent radiolabeled neutral amino acid unidirectional influx pathway attributable to known characteristics of B^0^AT1,^3–18,20–23,26,28,32^ as shown in Eadie–Hofstee plot of Figure 2. The B^0^AT1 transport activity data were obtained in the BBMVs according to eqn (4), as solved by nonlinear regression using the R package “investr” with objects of class “nls” employing the function:

(4) \begin{eqnarray*} {J^{\mathrm{ total}}} &=& \left\{ {\ J_{\mathrm{ max}}^{{\mathrm{ B^0}}\mathrm{ AT1}} \cdot \left[ S \right]\ /\ \left( {K_\mathrm{ m}^{{\mathrm{ B^0}}\mathrm{ AT1}} + \left[ S \right]} \right)} \right\} \\[5pt] &&+ \left\{ {\ J_{\mathrm{ max}}^{\mathrm{ Other}} \cdot \left[ S \right]\ /\ \left( {K_\mathrm{ m}^{\mathrm{ Other}} + \ \left[ S \right]} \right)} \right\} + \left\{ {P \cdot \left[ S \right]} \right\}, \end{eqnarray*}

where *J* represents influx initial rates, *J*_max_ is the maximal influx rate of a given transport carrier with its kinetics fitting the Michaelis–Menten relationship, [*S*] is the extravesicular radiolabeled l-alanine concentration (mm), *K*_m_ is the apparent Michaelis–Menten affinity constant for a given transport carrier, and *P* is the passive diffusion permeability coefficient. The computed value of *P* = 1.1 × 10^−7^ L/mg protein/5 s was also independently empirically verified by measuring total 0.1 mm [^3^H]-l-alanine uptake in media with K^+^ replacing Na^+^ and containing 100 mm unlabeled l-alanine and/or 100 mml-methionine. Based on nonlinear regression analyses, the B^0^AT1 component within the 95% CI (confidence interval) shown in Figure 2 yielded *J*^B^^0^^AT1^_max_ = 5.2 ± 0.4 nmol/mg protein/5 s, and *K*_m_^B^^0^^AT1^ = 6.9 ± 0.8 mml-alanine. Total influx included an apparent very minor additional non-B^0^AT1 saturable component, denoted “Other,” which was fitted in Figure 2 by *J*^Other^_max_ = 0.33 ± 0.06 nmol/mg protein/5 s, and *K*_m_^Other^ = 1.2 ± 0.3 mm (inset Figure 2B). This “Other” activity contributed <5% to maximal sodium-dependent uptake as compared with >95% of Na^+^-dependent active attributable to B^0^AT1. It could be speculated that “Other” might potentially represent systems ASCT2, SNAT2, or the [rBAT:b^0,+^AT1] heterodimer complex.^77^ However, unlike B^0^AT1, ASCT2 is an amino acid exchanger/antiporter^77^ that mechanistically would be principally unresponsive to the zero-*trans* initial rate unidirectional sodium-coupled uptake assay conditions employed in the present study (see the Methods section). Furthermore, ASCT2 is reportedly expressed in small intestine at levels ∼2.4% of B^0^AT1 expression,^26,78^ with ASCT2 prominence dominating ascending colon compared with small intestine. SNAT2^17^ is a highly unlikely candidate because it is primarily a basolateral membrane transport system that is expressed only transiently during the early development phase of life mainly in the neonatal duodenum, not in adult ileum as in our apical BBMV preparation. A [rBAT:b^0,+^AT1] heterodimer complex^17^ would run in reverse under the zero-*trans* initial uptake experimental conditions, thus likely precluding its activity. Thus “Other” activity was dropped from subsequent consideration in the ensuing analyses, and was discounted as a relevant factor in the present study.

**Figure 3.**
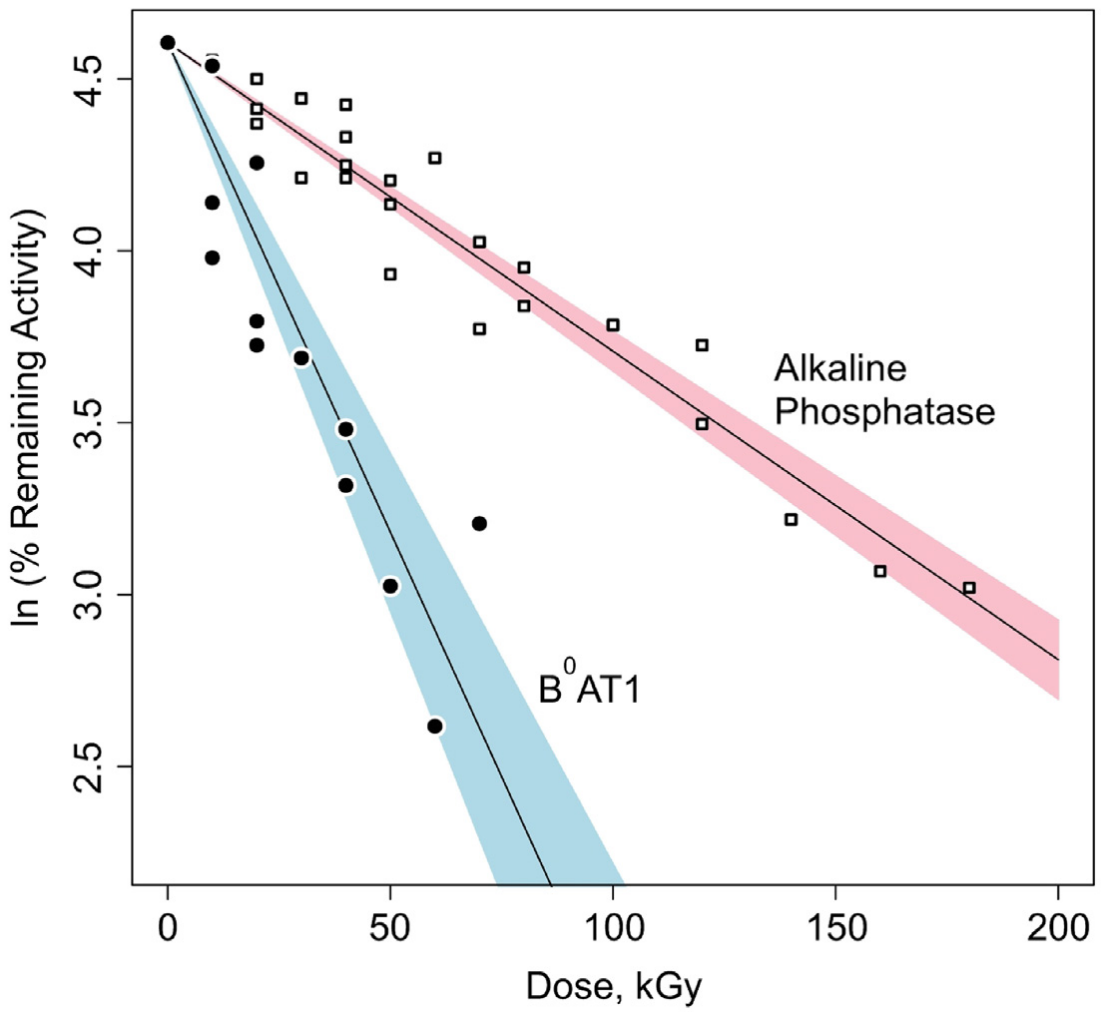
Radiation Inactivation of B^0^AT1 Transport and Alkaline Phosphatase Activities. At increasing electron irradiation doses, carrier-mediated sodium-dependent zero-*trans* unidirectional initial influx rates of [^3^H]-l-alanine or [^3^H]-L-serine uptake in intestinal BBMVs via B^0^AT1 (filled circles) were measured along with native alkaline phosphatase activity serving as the internal standard (open squares). Based on *ln* of % remaining activity at each dose compared with zero dose, nonlinear regression analyses (see the Methods section, eqns (2)–(4)) yielded target size mws. B^0^AT1 = 183.7 ± 16.8 kDa (blue: 95% CI for alanine and serine uptake, with D_37_ = 34.8 ± 3.3 kGy; *P* < .001). Alkaline phosphatase = 57.4 ± 1.8 kDa (pink: 95% CI for pNPP hydrolysis in Na^+^ media lacking K^+^, with D_37_ = 111.5 ± 3.5 kGy; *P* < .001).

Figure 3 shows B^0^AT1 transport activities and internal calibration standard alkaline phosphatase enzymatic activities remaining in reconstituted BBMVs exposed to increasing doses of high-energy electron irradiation. Based on eqns (1)–(3), nonlinear regression analyses of the radiation target theory relationships^57–70^ yielded target size mw and D_37_ value for B^0^AT1 = 183.7 ± 16.8 kDa (D_37_ = 34.8 ± 3.3 kGy; *P* < 0.001). For alkaline phosphatase hydrolysis of pNPP in Na^+^ media lacking K^+^, analyses yielded target size mw = 57.4 ± 1.8 kDa (D_37_ = 111.5 ± 3.5 kGy; *P* < 0.001).

**Figure 4.**
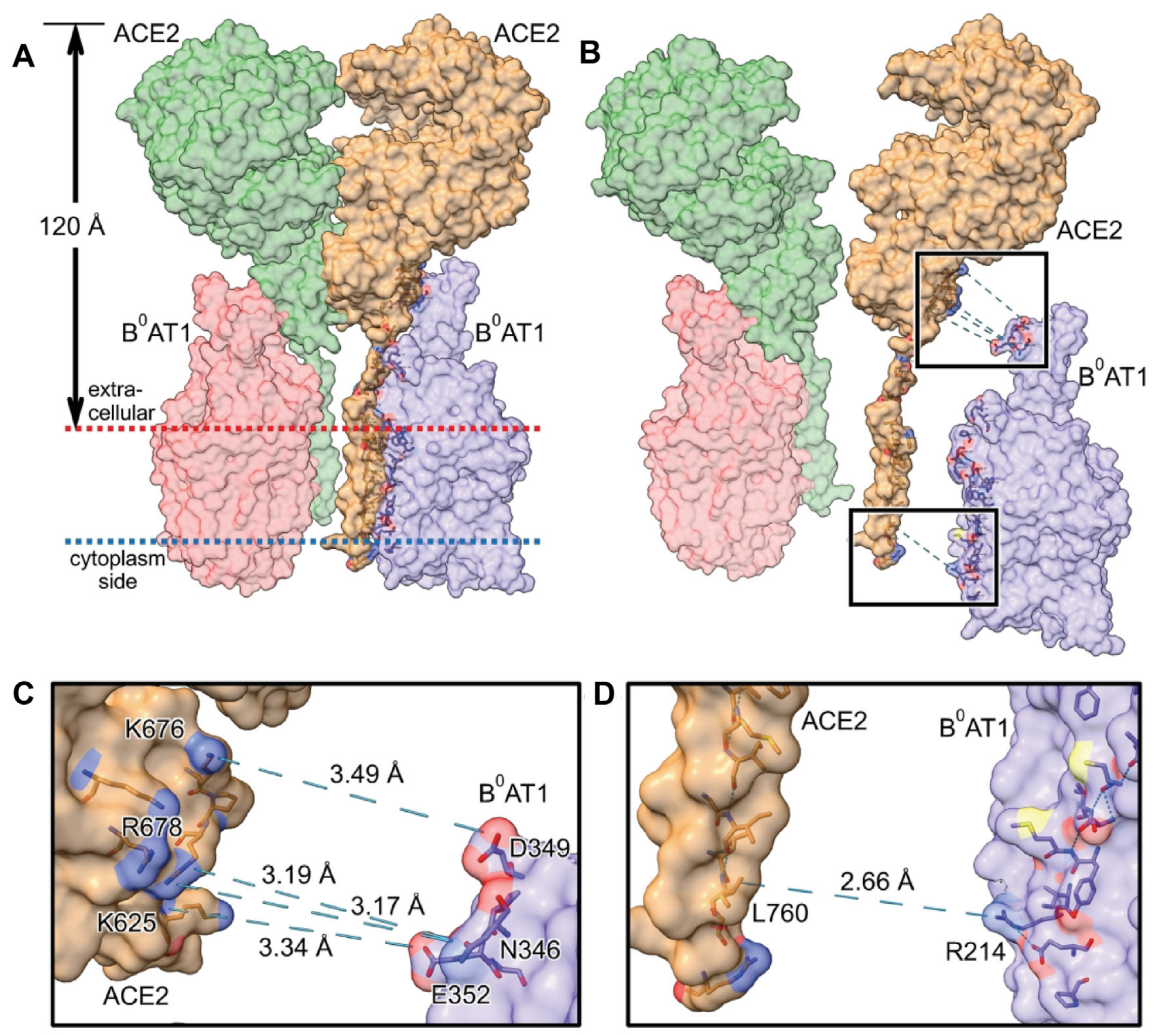
[ACE2:B^0^AT1]_2_ Dimer-of-Heterodimers Complex in Intestinal BBMVs. **(A)** PDB ID: 6M18^1^ is shown embedded in intestinal epithelial cell apical membrane surface. The [ACE2:B^0^AT1]_2_ hetero-4-mer complex total mass is 345.45 kDa assembled as a dimer of 2 [ACE2:B^0^AT1] heterodimers. Transmembrane hydrophobic residues of all chains anchor the complex within the membrane thickness of 30.2 Å (between red and blue dotted line boundaries), as determined using OPM database transmembrane server.^76^ The anchored structure protrudes 120 Å from the membrane surface. **(B)** Exploded view of panel A emphasizing contact bonds for 1 of the 2 internal heterodimer [ACE2:B^0^AT1] bonding interfaces in the regions of extracellular milieu (upper box) and membrane anchors (lower box). Interface contact residues were the same whether for heterodimer ACE2_chain_B paired with B^0^AT1_chain_A (shown exploded), or heterodimer ACE2_chain_D paired with B^0^AT1_chain_C. **(C)** Enlarged exploded view of upper box of panel B, showing bond distances between contact residues. **(D)** Enlarged exploded view of lower box of panel B, showing bond distance between contact residues. Key: B^0^AT1_chain_A, purple; B^0^AT1_chain_C, pink; ACE2_chain_B, tan; ACE2_chain_D, green.

Atomic coordinates for PDB ID: 6M18^1^ represent the thermodynamically favored assembly of the dimer-of-heterodimers complex putatively embedded in the intestinal epithelial cell apical brush border membrane surface. Figure 4 shows this [ACE2:B^0^AT1]_2_ quaternary complex total mw = 345.45 kDa assembled as a dimer of 2 [ACE2:B^0^AT1] heterodimers. Employing the OPM database transmembrane server,^76^ we calculated that transmembrane hydrophobic residues of all chains secure the complex within a BBMV membrane thickness of 30.2 Å, and that the anchored structure protrudes 120 Å from the membrane surface (Figure 4A). Figure 4B, the exploded view of panel 4A, emphasizes the zones of contact bonds connecting the subunits of the internal heterodimer [ACE2:B^0^AT1] interface residues in the regions of extracellular milieu (upper box) and membrane anchors (lower box); for graphic simplicity, only the right side [ACE2:B^0^AT1] exploded pairing is shown (tan color ACE2_chain_B with purple B^0^AT1_chain_A), although the same relationships hold for the Figure 4B left side unexploded pairing of green ACE2_chain_D complexed with pink B^0^AT1_chain_C. Employing PDBePISA, ChimeraX, PyMOL, and OPM,^72–74,76^ we computed the interface contact amino acid residues as being the same whether for heterodimer ACE2_chain_B paired with B^0^AT1_chain_A (shown exploded), or for heterodimer ACE2_chain_D paired with B^0^AT1_chain_C. Figure 4C is an enlarged exploded view of the upper box of panel 4B, showing bond distances between specific contact residues. Figure 4D shows an enlarged exploded view of lower box of panel 4B, revealing bond distance measured between specified contact residues. The interface bonding computations are summarized in the data of Table 1. These results indicate that within the [ACE2:B^0^AT1]_2_ dimer-of-heterodimers complex, each of the separate heterodimer [ACE2:B^0^AT1] chain pairing combinations yielded bonds with statistically significant (*P* = .037 for pairing of [ACE2_chain_B:B^0^AT1_chain_A]; and *P* = .040 for pairing of [ACE2_chain_D:B^0^AT1_chain_C]) negative free energy minimization Δ^i^G = −20.8 kcal/mol over an interface surface area of 1260.8 Å^2^, unlike the nonsignificant difference in the homodimer bond pairing of ACE2_chain_A:ACE2_chain_D residue contacts (*P* = .935; positive Δ^i^G = +3.8 kcal/mol).

**Table 1.** Interface Bonds within the [ACE2:B^0^AT1]_2_ Dimer-of-Heterodimers Complex

Chain Pairing	Δ^i^G (kcal/mol)	*P*-value	Interface Surface Area (Å^2^)	Hydrogen Bonds Between Contact Residues (Distance Å)
[ACE2:B^0^AT1] (chain_B:chain_A)	−20.8	.037	1260.8	ACE2_LEU760/B^0^AT1_ARG214 (2.66 Å)ACE2_ARG678/B^0^AT1_ASN346 (3.17 Å)ACE2_ARG678/B^0^AT1_ASN346 (3.19 Å)ACE2_LYS625/B^0^AT1_GLU352 (3.34 Å)ACE2_LYS676/B^0^AT1_ASP349 (3.49 Å)
[ACE2:ACE2] (chain_B:chain_D)	+3.8	.935	1276.9	N/A
[B^0^AT1:B^0^AT1] (chain_A:chain_C)	N/A	N/A	null	N/A
[ACE2:B^0^AT1] (chain_D:chain_C)	−20.8	.040	1263.1	ACE2_LEU760/B^0^AT1_ARG214 (2.66 Å)ACE2_ARG678/B^0^AT1_ASN346 (3.17 Å)ACE2_ARG678/B^0^AT1_ASN346 (3.19 Å)ACE2_LYS625/B^0^AT1_GLU352 (3.34 Å)ACE2_LYS676/B^0^AT1_ASP349 (3.49 Å)

Solvation-free energies were calculated for each isolated chain, and also for the interfaces between contact residues of chain combinations within the [ACE2:B^0^AT1]_2_ dimer-of-heterodimers complex described in Figure 4. The Δ^i^G values represent solvation-free energy gain (kcal/mol) upon formation of a given interface, with *P* ≤ .05 representing statistical significance. Shown are the distances between specific residues responsible for interface contact hydrogen bonds between paired chains shown in Figure 4. There were null interactions between the B^0^AT1 chains.

**Figure 5.**
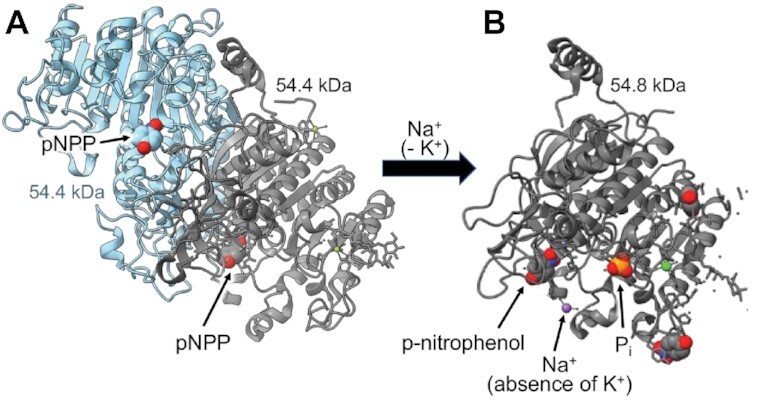
Alkaline Phosphatase (EC 3.1.3.1) With and Without Na^+^. **(A)** Rat intestine alkaline phosphatase homodimer assembled as two 54.4 kDa monomers without Na^+^ (PDB ID: 4KJG), shown with pNPP substrate in each binding site. **(B)** Alkaline phosphatase 54.8 kDa monomer activity assayed under conditions of Na^+^ ion (purple) in the absence of K^+^ (PDB ID: 3MK1), shown with pH 9.8 reaction products *p*-nitrophenol and inorganic phosphate (P_i_).

Intestinal-type alkaline phosphatase (EC 3.1.3.1) was chosen as the radiation inactivation target size internal calibration standard (Figure 3; target size mw = 57.4 ± 1.8 kDa), grounded on various mammalian orthologs exhibiting the same fundamental structural arrangement running as a single ∼55 kDa monomer Western blot band.^79^ It has been previously demonstrated^62^ that the radiation inactivation target size mw of intestinal alkaline phosphatase monomer can be identified independent from the homodimer state when the postirradiation enzyme activity is assayed under conditions of using the Na^+^ salt of pNPP substrate hydrolysis in the absence of K^+^ at alkaline pH,^62,66^ as described earlier in the Methods section. Rat intestinal-type alkaline phosphatase atomic coordinates (PDB ID: 4KJG) indicate a homodimer assembly of 2 identical noncovalently associated independent 54.4 kDa monomer chains in the absence of Na^+^, as shown in Figure 5. Further in Figure 5, the effect of binding Na^+^ ion in the absence of K^+^ is revealed as shown by the 54.8 kDa monomer structure from atomic coordinates of human alkaline phosphatase PDB ID: 3MK1, with release of *p*-nitrophenol product.^80^

## Discussion

The main finding of this study is that sodium-dependent carrier-mediated B^0^AT1 activity *in situ*in small intestinal enterocyte purified apical BBMVs occurs via an apparent physiological “functional unit” of target size mw = 183.7 ± 16.8 kDa representing a thermodynamically stabilized [ACE2:B^0^AT1] heterodimer, determined by high-energy electron radiation inactivation analysis. This finding is consistent with predictions in the literature grounded in prior biochemical, immunohistochemical, molecular modeling, and cryo-EM techniques. Two of these heterodimer functional units behave within the physical structure of an [ACE2:B^0^AT1]_2_ dimer-of-heterodimers 4-mer complex, with PDB ID: 6M18 atomic coordinates measured by Yan et al.^1^ Notably, these data are consistent with our prior molecular docking modeling^55,56^ and gut–lung axis studies,^33,34,81^ and prescient antecedent literature review^17^ that putatively implicated the B^0^AT1 subunit as a major player with ACE2 in SARS-CoV-2 gastrointestinal tropism in COVID-19.

Previous experimental evidence demonstrated that posttranslational SLC6A19 gene expression of B^0^AT1 and its sodium-dependent neutral amino acid transporter activity obligatorily engages the accessory protein ACE2 as its chaperone for intracellular trafficking to epithelial cell apical brush border membranes, whereby the mature B^0^AT1 protein subunit colocalizes with ACE2 within the membrane.^18,20,22,24–28,82–85^ Pharmacologic manipulation of ACE2 expression demonstrated concomitant parallel changes in B^0^AT1 amino acid transporter protein expression and uptake activity^18,20,22,24–28,82–85^; however, the converse does not hold, such that ACE2 can be expressed independent of trafficking B^0^AT1.

The individual molecular masses of B^0^AT1, ACE2, and [ACE2:B^0^AT1]_2_ physical structures have each been determined previously based on molecular biology, biochemistry, cell transfection/expression, tissue immunofluorescence microscopy colocalization, and epithelial membrane isolation techniques.^18,20,22,24–28,82–84^ Western blots yielded a single band for each component, reflecting the appropriate molecular weights of each individual cloned monomer (denaturing conditions) or aggregate multimer complex (native gel conditions). The B^0^AT1 monomer subunit band on SDS-PAGE^23,86,87^ is ∼75 kDa, with predicted mw = 71.2 kD from 634 amino acids expressed by the SLC6A19 gene (accession NP_001034811.1). ACE2 monomer single bands generally range from ∼110 kDa (glycosylated) to ∼92 kDa (deglycosylated), with predicted mw = 92.5 kDa from 805 amino acids expressed by the ACE2 gene (accession XP_002719891.1).^88–91^ In mouse intestinal purified brush border membranes, B^0^AT1 and ACE2 coimmunoprecipitation coupled with digitonin native PAGE yielded a band at 376 or 488 kDa, representing the intact [ACE2:B^0^AT1]_2_ dimer-of-heterodimers 4-mer complex.^18^ Collectively, these biochemical findings are consistent with the 2.9 Å resolution cryo-EM (PDB ID: 6M18) atomic structure^1^ mw ∼345 kDa (replete with hydrogen atoms) for 2 [ACE2:B^0^AT1] heterodimers assembled as a [ACE2:B^0^AT1]_2_ dimer-of-heterodimers ternary complex shown in Figure 4. As further shown in Figure 4A, the membrane-anchored [ACE2:B^0^AT1]_2_ complex protrudes 120 Å outward from the extracellular surface. This is consistent with the well-known phenomenon reported for a wide variety of integral membrane-bound protein multimer ectodomains anchored by lipid rafts in epithelial cell membranes,^1,17,76,92^ and is in agreement with the results in Figure 1B and C electron micrographs showing 100–150 Å protruding knobs on the BBMVs employed in the present study.

While such biochemical and physical techniques are useful to identify purified individual polypeptides and their physical characteristics, the unique value of radiation inactivation analysis is to reveal structure–function relationships and biological behaviors especially *in situ* in oligomeric protein assemblies of any form—whether crude samples, intact cells, membranes, or purified molecules. Ionizing radiation inactivation target theory has been used extensively to assess the physiological behavior “functional unit” molecular masses of a diverse variety of complex multi-subunit oligomeric polypeptide structures residing *in situ* in biological systems, such as channels, transporters, enzymes, and receptors,^59–70^ including our prior work with intestinal integral membrane-bound proteins in BBMVs.^57,58^ The literature is replete with evidence of radiation inactivation accurately assigning known biological activities as a “functional unit” whether as a single polypeptide or as an oligomeric assembly of many individual polypeptide subunits.^59–70^ The technique exploits the loss of measured biological activity surviving a random hit by a high-energy electron from a linear accelerator, with the probability of being knocked out by deposition of the electron's 60 eV (1500 kcal/mol) ionizing energy directly correlated with the mw “target size” of the functioning entity, as described in the Methods section and extensively discussed elsewhere.^59–70^ In the case of biological activity of a multimer composed of subunits, a single electron hitting any one of the subunit members within the collective assembly will completely abolish functional activity as the consequence of transferring its ionizing energy to other subunits of the complex via bonds of contact interface amino acid residues.^59–70^ Thus, for a heterodimer with subunits paired by 1 or more bond of interface contact residues, and in accordance with radiation inactivation target theory,^59–70^ an electron direct hit to either one of the subunits will nullify biological activity, even if only one of the subunit entities is responsible for the actual biological activity.

The data of Figure 3 fit the simple exponential relationship of eqns (1)–(3) for the inactivation of membrane *in situ* B^0^AT1 transport activity. The computed values in Table 1 summarizing the structures of Figure 4 indicate that [ACE2:B^0^AT1] heterodimer pairings are thermodynamically stabilized (Δ^i^G = −20.8 kcal/mol) via interface contact bonds 2.66–3.49 Å involving 5 specific residue pairings within the hetero 4-mer complex. However, this is in contrast to atomic modeling attempts (Table 1 and Figure 4) to examine [B^0^AT1:B^0^AT1] or [ACE2:ACE2] homodimer pairings that each lack residues with bonds able to transfer electron hit energy into the adjoining subunits (Δ^i^G = +3.8 kcal/mol in the case of [ACE2:ACE2]; and null interfacings between the B^0^AT1 subunits). Thus, a high-energy electron direct hit to any ACE2 subunit will transfer its energy to a B^0^AT1 subunit, resulting in annihilating measurable B^0^AT1 transport activity. Based on eqns (2) and (3), the above arguments collectively indicate that the high-energy electron irradiation “sees” a functional unit target mw ∼184 kDa for B^0^AT1 transport activity, which is consistent with radiation target theory describing a multimeric functional unit^59–70^ composed of the [ACE2:B^0^AT1] heterodimer.

The radiation inactivation target size results (Figure 3) were validated by internal calibration exploiting endogenous alkaline phosphatase activity in the reconstituted BBMVs. As shown in Figures 3 and 5 for the K^+^-independent activity of pNPP hydrolysis assayed in the presence of Na^+^, the data revealed the internal alkaline phosphatase radiation target mw = 57.4 ± 1.8 kDa, consistent with prior studies predicting ∼55 kDa monomer subunits on Western blots.^62,66,79,80^

The present study employed native intestinal BBMV membranes. We posit that it would be beneficial to extend such studies to include future explorations of drug interactions and effects of membrane lipid raft stabilization relating to SARS-CoV-2 tropism in the intestine, in contrast to events in lung pneumocytes that lack B^0^AT1. Such tools include, for example: (1) the recent expression of B^0^AT1 in bacteria^93^; (2) HEK293 cells' coexpression of ACE2 with B^0^AT1^1^ as exploited by Pfizer/BioNTech to screen their mRNA vaccine candidates against SARS-CoV-2^2^; and (3) the recent discoveries of nimesulide^94^ and cinromide^95^ as inhibitors of B^0^AT1. Coexpression evidence suggests that the small intestinal BBMV SIT1 (SLC6A20), representing the IMINO transport system serving proline uptake originally described by us,^17,96,97^ also functionally partners with epithelial membrane ACE2.^26,98^ Thus, we posit that it would be beneficial to pursue the atomic structural interactions, functional relationships, and effects of targeted drugs engaging SITS1 relating to COVID-19 in the manner analogous to B^0^AT1 with ACE2. Furthermore, such future experimental pursuits would bear fruit relating to our in silico studies^33,34,55,56^ that have implicated a role for B^0^AT1 and SITS1 in sterically governing the role of intestinal membrane proteinase TMPRSS2 and ADAM17 as mediators of ACE2-dependent intestinal SARS-CoV-2 infection and gut inflammasome induction.

In conclusion, high-energy electron radiation inactivation analysis was used to determine that B^0^AT1 transport activity occurs via the [ACE2:B^0^AT1] heterodimer functional unit housed within the physical structure of the [ACE2:B^0^AT1]_2_ dimer-of-heterodimers quaternary complex embedded in the apical brush border membranes of small intestinal enterocytes. It is noteworthy that SARS-CoV-2 virus hijacks ACE2 as its receptor and entry point of infecting cells, and further that the small intestine is the body's site of greatest magnitude of expression of both B^0^AT1 and ACE2.^33,34^ Thus, the [ACE2:B^0^AT1] heterodimer functional unit is important for gut lumen activities (1) relating to pleiotropic native physiological roles in amino nitrogen metabolism of nutritive and bioactive peptides, (2) in local gut mucosa renin–angiotensin system regulating absorption of sodium and organic nutrients, and (3) as central to steering SARS-CoV-2 tropism in the GI tract with attending GI shedding of SARS-CoV-2 particles and clinical symptomology in about half of COVID-19 patients,^17,25,33,34,42,55,56,81^ including bacteremic inflammation of gut dysbiosis origin in COVID-19 patients.^99^ These findings enhance our understanding of gut pathophysiology, thereby contributing to future translational experiments designed to treat or mitigate COVID-19 variant outbreaks and/or GI symptom persistence in long-haul PASC.

## Data Availability

The data underlying this article will be shared on reasonable request to the corresponding author.
